# Awareness and Attitude of Undergraduate Medical Students towards 2019-novel Corona virus

**DOI:** 10.12669/pjms.36.COVID19-S4.2636

**Published:** 2020-05

**Authors:** Azal Ikhlaq, Hunniya Bint-E-Riaz, Imtiaz Bashir, Farhat Ijaz

**Affiliations:** 1 Azal Ikhlaq, 2^nd^ year MBBS Medical Student, CMH LMC & IOD, Lahore, Pakistan; 2 Hunniya Bint E Riaz, 2^nd^ year MBBS Medical Student, CMH LMC & IOD, Lahore, Pakistan; 3 Imtiaz Bashir, 2^nd^ year MBBS Medical Student, CMH LMC & IOD, Lahore, Pakistan; 4 Dr. Farhat Ijaz, MPhil Department of Physiology CMH LMC & IOD, Lahore, Pakistan

**Keywords:** Awareness, Attitude, Medical students, 2019-nCoV

## Abstract

**Objective:**

To assess the knowledge, awareness and attitudes of medical students towards recently discovered coronavirus disease-19 (COVID-19).

**Methods:**

This was a cross sectional study conducted on medical students in CMH Lahore Medical College,(LMC), Institute of Dentistry (IOD). A questionnaire containing demographic information, 14 knowledge and eight attitude items was completed by 384 participants.

**Results:**

Overall, >90% people were aware about the etiology, mode of transmission and possible symptoms; however, very few of them knew about the in-depth details. Knowledge score revealed that 80% of participants had sufficient knowledge about coronavirus. MBBS students and nursing Students had significantly better knowledge in comparison with other students. In terms of attitude, >80% of students showed positive attitudes among which the nursing students were dominant.

**Conclusion:**

The medical students of CMH LMC showed a satisfactory level of awareness and attitudes towards COVID-19 with an obvious difference with regard to disciplines. More educational efforts with periodic educational interventions are still needed about the current pandemic.

## INTRODUCTION

COVID-19 has become a pandemic these days and it is a topic of high public concern and medical students are directly or indirectly related to it. Many people reported to different hospital in Wuhan, China with the complaints of fever, headache, shortness of breath, malaise and dry cough in mid-December 2019. They were admitted as the cases of atypical pneumonia. Some of them developed complications as the disease progressed. They developed respiratory failure and were given ventilatory support.^1^

On December 12, 2019, the first case of this type of pneumonia was detected. The possibilities of other coronaviruses, influenza and other such diseases were ruled out by laboratory diagnostics. It was announced by the Chinese authorities on January 7, 2020 that a new strain of coronavirus was discovered and isolated in those patients. World Health Organization (WHO) provisionally named this virus as 2019 novel coronavirus (2019-nCoV) on January 12, 2020. The disease caused by this virus was termed as Coronavirus Diseases of 2019 (COVID-19) on Feb 11, 2020 and the virus was renamed as Severe Acute Respiratory Syndrome 2 Coronavirus (SARS-2 coronavirus).^2^

As of now (March 16,2020), there is a total of 142,539 reported cases of COVID-19 globally, with 5393 deaths. The pandemic has spread to many countries with extension to 13 more countries in just past 24 hours. There are 22 confirmed cases of coronavirus in Pakistan out of which one patient has died. Most patients have had a history of travelling abroad recently.^3^

2019-nCoV belongs to the family Coronaviridae (commonly called Coronavirus). Coronaviruses are enveloped RNA viruses known to cause respiratory, hepatic and neurological diseases.^4^ They have a wide distribution among birds, mammals, and humans.^5^ Six species of coronaviruses have been identified to cause disease in human. Out of these six, four --- 229E, OC43, NL63, and HKU1 --- are quite prevalent and cause symptoms of common cold in immunocompetent people.^6^

Coronavirus differs from other enveloped viruses in its replicative cycle by the fact that its envelope is derived from the endoplasmic reticulum of the host cell not from the plasma membrane. This may add to its pathogenicity.^7^ Envelope (E) proteins, Membrane (M) proteins and Spike (S) proteins have been identified in coronavirus.^8^ S-proteins have been found to have a role in the recognition and attachment of the virus to the Angiotensin Converting Enzyme 2 (ACE-2) receptors of the human epithelial cells in the respiratory mucosa.^9^ These features of COVID-19 bear resemblance with the infections caused by previously known Middle East Respiratory Syndrome (MERS) and Severe Acute Respiratory Syndrome (SARS) coronaviruses.^10^

The mode of transmission of coronaviruses is still ambiguous and not well established. However, it is believed that the virus spreads through respiratory aerosol by coughing and sneezing of the infected person, or via close personal contact. Infection may spread by touching contaminated objects.^11^

As far as the treatment of COVID-19 is concerned, there is no specific antiviral medicine or vaccine to treat or prevent the disease. Treatment for symptomatic relief can be done. Possible vaccine and medications are under trial.^12^

Medical Students are directly or indirectly related to such epidemics. So, they must have a higher level of knowledge and better attitude towards such diseases. That’s why our objective was to assess the level of awareness and attitude of medical students towards this disease. Till now, no such study regarding 2019-nCoV has been done.

## METHODS

A cross-sectional study based on administration of questionnaire was carried out at Combined Military Hospital, Lahore Medical College and Institute of Dentistry, Lahore in February 2020 after approval from ethical review committee. The population under study consisted of medical students including those studying MBBS, BDS, Nursing and Allied Health Sciences. Undergraduate Medical Students and those who were willing to participate were included.

The sample size was calculated (through RaoSoft, using the formula, n = Z2*P (1-P)/m2) to be 384 with a 95% confidence interval and 5% error margin. Using random sampling, 384 participants were taken into account subsequent to receiving informed written consent. Any ambiguities of participants regarding the questionnaire were addressed in detail. The questionnaire consisted of four parts: 1. Demographic profile 2. Information Regarding Source of Knowledge 3. 11 awareness questions 4. Eight attitude-based questions

All the data were entered into SPSS software version 25.0 (IBM, Armonk, NY) for analysis and validation. The qualitative variables were presented in the form of frequencies and percentages. Information was expressed in the form of tables.

## RESULTS

Total of 384 medical students completed the questionnaire. The participants ranged from an age of 18 to 28 years. They were divided into two groups. Group A having age ranging from 18-21 and Group B having age ranging from 22-25. Total of 77.9% participants fall in Group A and 22.1 % in Group B. The baseline characteristics of the respondents is shown in Table-I.

**Table-I:** Baseline characteristics of the participants, 2020.

Characteristic	Participants (n=384) No. (%)
***Sex***	
Male	159 (41.4%)
Female	225 (58.6%)
***Age***	
Group A (18-21 years)	299 (77.9%)
Group B (22-25 years)	85 (22.1%)
***Discipline***	
MBBS	251 (65.4%)
BDS	43 (11.2%)
Nursing	47 (12.2%)
Allied Health Sciences	43 (11.2%)
***Year of Study***	
1^st^ Year	100 (26%)
2^nd^ Year	148 (38.5%)
3^rd^ Year	85 (22.1%)
4^th^ Year	26 (6.8%)
5^th^ Year	25 (6.5%)

About 80% of the participants discerned that they had acquaintance about coronavirus. Table-II. Major sources of information about coronavirus were social media (53.1%) and Television (18%).

**Table-II:** Perception about Knowledge and Source of knowledge of Coronavirus of the respondents.

Question	Answer n= 384 No. (%)
***Do you know about Coronavirus?***	
Yes	307 (79.9)
No	52 (13.5)
Don’t Know	25 (6.5)
***If yes, What is source of knowledge?***	
Ministry of Health Website	26 (6.8)
Social Media	204 (53.1)
Newspaper	15 (3.9)
Television	69 (18.0)
Others	6 (1.6)

The items level of awareness about coronavirus among medical students is shown in Table-III. Majority were aware of the viral nature of the infection(97.4%) and well-acquainted with the mode of transmission of the infection (97.4%).While 91.9% knew that fever along with cough, and shortness of breath are the signs and symptoms of the disease

**Table-III:** Current Status of Knowledge of medical undergraduates about coronavirus.

Question (Correct Answer)	Correct Answer No. (%)
1. Coronavirus is a viral infection. (Yes)	374 (97.4)
2. Coronavirus is transmitted by close contact with infected person or animal	374 (97.4)
3. Fever, Cough and Shortness of Breath are symptoms of coronavirus. (Yes)	353 (91.9)
4. Diarrhea is a possible symptom of coronavirus. (Yes)	140 (37.5)
5. The incubation period is 2-4 weeks. (Yes)	250 (65.1)
6. Coronavirus vaccine is available in markets. (No)	298 (77.6)
7. Antibiotics are the first line treatment (No)	243 (63.3)
8. Washing hands with soap and water can help in prevention of disease transmission. (Yes)	326 (84.9)
9. Patients with underlying chronic diseases are at a higher risk of infection. (Yes)	324 (84.4)
10. Health care workers are at a higher risk of infection. (Yes)	353 (91.9)
11. Coronavirus can be fatal. (Yes)	346 (90.1)

The attitude of the undergraduate medical students towards coronavirus is shown in Table-IV. There were 56% participants who were worried that one of their family members might get the infection.

**Table-IV:** Attitude of Undergraduate Medical Students towards coronavirus.

Item (Correct Answer)	Response No. %
1. Are you worried one of your family members may get an infection? (Yes)	215 (56)
2. Transmission of coronavirus can be prevented by using standard and isolation precautions given by WHOb. (Yes)	347 (90.4)
3. Prevalence of coronavirus can be reduced by active participation of health care workers in hospital infection control program. (Yes)	346 (90.1)
4. If a coronavirus vaccine were available, would you have it? (Yes)	331 (86.2)
5. Intensive treatment should be given to diagnosed patients. (Yes)	351 (91.4)
6. Health care workers must avail themselves of all information about the virus.	353 (91.3)
7. Is available information in the Pakistani society sufficient? (Yes)	80 (21.8)
8. Are the government institutions capable of combating the epidemic of coronavirus? (Yes)	76 (20.8)

## DISCUSSION

We found that more than half percentage of people relied on social media as a source of information. This is in accordance with previously done KAP studies on a previous strain of coronavirus (MERS-CoV).^13^-^14^ Social media as a source of information is a two-way street. On one hand, it is cost-effective, wide-dispersal and easily accessible source while on the other hand, it spread fake information. Fake news and false information can have devastating effects on the society.^15^-^16^ It puts an increased liability on health care authorities to enhance the availability and approachability of required authentic information by using varied and effective means of communication. Medical students should carefully evaluate of coronavirus related awareness materials before sharing or applying it.

A healthy relationship between the availability of information in the media and the level of awareness among medical undergraduates is established in the findings of this study. Examples elaborating this relationship are awareness of about 85% of the participants about the precautionary actions, 95.3% about the mode of spread, 97.4% about infecting agent, and of 91.9% participants about the presenting complaints. These results concord with the conclusions of previous surveys.^17^-^18^ Only a very few respondents knew about diarrhea as a symptom of coronavirus. One reason might be that diarrhea is a less common symptom of the disease and that’s why it is not well illustrated on media and as the major source of knowledge of medical students about coronavirus is social media and television so they did not know about it. On the other hand, large number of respondents were aware of incubation period. This shows a better awareness level of medical students about coronavirus than that showed for other strains of coronaviruses in previously conducted surveys.^17^

In this study, 79.9% medical students had enough knowledge about coronavirus. The results point out the need for better and systemized efforts to increase the level of awareness among medical students. As anticipated, discipline and year of study was appreciably linked with higher levels of information. Students belonging to MBBS and nursing and having a higher year of study showed higher level of knowledge and awareness. Previous studies on other strains of coronavirus showed an association between age and level of knowledge.^11^,^13^ Educational program should be intended to target the professions with established lower level of awareness, i.e. in our study this turns out to be dental and Allied health sciences students who were in their initial year of study.

Most of the respondents had a generally encouraging outlook towards contribution in infection control campaigns and awareness programs. However, it is important to note that 44% respondents were not worried that one of their family members might get an infection. There is a need of proper awareness about the gravity of the condition by using accessible and potent means of information. A large number of respondents did not believe in the ability of government to combat the imminent epidemic. The reason might be a communication gap between the masses and the government. There is a dire need for health care authorities to improve their communication with the masses and to increase the confidence of a common man in health control programs. This can be done by social media campaigns, seminars, awareness advertisements and improving the curriculum.

A negative attitude towards immunization was shown by 14% of the respondents. About 91.3% of the participants believed that maximum information available about the disease must be availed. Majority agreed with the grimness of complications by agreeing with the intensive care of the diagnosed patients. These findings are concurrent with previous surveys on other strains of coronavirus.^13^,^17^,^18^

As per WHO recommendations, Pakistani government has directed the health care authorities to establish enlightening crusade aimed at awareness about the prevention, treatment and symptoms of coronavirus.^19^ However, greater endorsement is needed for health care workers especially and all medical students generally to refer to the authentic sources for awareness and knowledge about coronavirus.

### Limitations of the study

The study was done in a single medical institute in Lahore, Punjab region of Pakistan, thus the results shown here may not be applicable to other areas of the country. Conduction of extensive studies from other regions is important to investigate awareness and attitude of students’ nationwide level.

## CONCLUSION

The undergraduate medical students in CMH Lahore showed a satisfactory level of awareness and positive attitudes towards coronavirus with an obvious difference in awareness level between various disciplines. Better educational efforts with effective techniques are pointed to further increase the level of awareness and to suffice for the shortcomings. More efforts should be directed at dentists, Allied health Sciences specially and to all medical students generally. Health Care Authorities should be more involved in the process of education about the pandemic.

### Authors Contribution

**IB:** Conceived, designed and did statistical analysis & editing of manuscript.

**AI, HBR, IB:** Did data collection and manuscript writing.

**FI:** Did overall supervision, final drafting, revision and final approval of manuscript.
